# Micronutrient Food Supplements in Patients with Gastro-Intestinal and Hepatic Cancers

**DOI:** 10.3390/ijms22158014

**Published:** 2021-07-27

**Authors:** Waqas Alam, Hammad Ullah, Cristina Santarcangelo, Alessandro Di Minno, Haroon Khan, Maria Daglia, Carla Renata Arciola

**Affiliations:** 1 Department of Pharmacy, Abdul Wali Khan University, Mardan 23200, Pakistan; waqasalamyousafzai@gmail.com (W.A.); haroonkhan@awkum.edu.pk (H.K.); 2 Department of Pharmacy, University of Naples Federico II, 80131 Naples, Italy; hammad.ullah@unina.it (H.U.); cristina.santarcangelo@unina.it (C.S.); alessandro.diminno@unina.it (A.D.M.); 3 CEINGE-Biotecnologie Avanzate, Via Gaetano Salvatore 486, 80145 Naples, Italy; 4 International Research Center for Food Nutrition and Safety, Jiangsu University, Zhenjiang 212013, China; 5 Laboratorio di Patologia delle Infezioni Associate all’Impianto, IRCCS Istituto Ortopedico Rizzoli, Via di Barbiano 1/10, 40136 Bologna, Italy; 6 Department of Experimental, Diagnostic and Specialty Medicine, University of Bologna, Via San Giacomo 14, 40136 Bologna, Italy

**Keywords:** micronutrients, gastrointestinal cancer, hepatic cancer, cancer therapy, molecular mechanisms

## Abstract

Colorectal carcinogenesis is the second most common cause of mortality across all types of malignancies, followed by hepatic and stomach cancers. Chemotherapy and radiotherapy are key approaches to treating cancer patients, but these carry major concerns, such as a high risk of side effects, poor accessibility, and the non-selective nature of chemotherapeutics. A number of natural products have been identified as countering various forms of cancer with fewer side effects. The potential impact of vitamins and minerals on long-term health, cognition, healthy development, bone formation, and aging has been supported by experimental and epidemiological studies. Successful treatment may thus be highly influenced by the nutritional status of patients. An insufficient diet could lead to detrimental effects on immune status and tolerance to treatment, affecting the ability of chemotherapy to destroy cancerous cells. In recent decades, most cancer patients have been taking vitamins and minerals to improve standard therapy and/or to decrease the undesirable side effects of the treatment together with the underlying disease. On the other hand, taking dietary supplements during cancer therapy may affect the effectiveness of chemotherapy. Thus, micronutrients in complementary oncology must be selected appropriately and should be taken at the right time. Here, the potential impact of micronutrients on gastro-intestinal and hepatic cancers is explored and their molecular targets are laid down.

## 1. Introduction

The World Health Organization (WHO) most recently reported cancer as a leading cause of death, with 10 million worldwide deaths in 2020. Of the various types of cancer, colon and rectum cancer was the second most common cause of death in 2020 with 935,000 deaths per year, followed by hepatic cancer (830,000 deaths) as third, and stomach cancer (769,000 deaths) as the fourth most common cause of mortality [1]. In addition to its negative health impact and increased mortality rate, the annual economic cost of cancer is also increasing significantly, estimated by the International Agency for Research on Cancer in 2010 to be US 1.16 trillion [2]. Adenocarcinoma is the most common type of gastric cancer, and the important factors associated with the incidence and prevalence of gastric cancers include an unhealthy diet, alcohol consumption, smoking, *Helicobacter pylori* infections, and pernicious anemia [3,4]. Risk factors associated with colorectal cancers include obesity, smoking, sedentary lifestyle, low fiber diets, red and processed meat, and inflammatory bowel disease (Crohn’s disease and ulcerative colitis) [5,6,7]. Hepatic cancers, also known as hepatocellular carcinoma (HCC) or hepatoma, typically result from hepatitis B or C infections, cirrhosis, and chronic alcoholism [8,9]. Metabolic dysregulations such as nonalcoholic steatohepatitis and type 2 diabetes, probably aided by obesity, may also provide a road map for the pathogenesis of HCC [10,11].

Chemotherapy and radiotherapy, alone or in combination, have, for many years, been considered to be the key approaches for the treatment of patients with various types of cancer. However, conventional chemotherapies carry some major concerns that may outweigh their therapeutic benefits in certain cases, such as an increased incidence of side effects, poor accessibility to tumor tissues, and the non-selective nature of chemotherapeutic drugs [12,13]. Moreover, conventional chemotherapy is capable only of the fractional killing of cells, i.e., a number of cancerous cells die with each treatment, and thus repeated doses may be administered to effectively reduce the tumor size [14]. Fast-dividing cells, such as cells of the hematopoietic and gastrointestinal (GI) systems, are the most affected by chemotherapeutic drugs and are thus most prone to side effects. GI distress (nausea, vomiting, diarrhea, anorexia, constipation, and abdominal cramps) [15,16], alopecia [17], immunosuppression [18], myelosuppression [19], anemia [20], neutropenic enterocolitis [21], infertility [22], teratogenicity [22], secondary neoplasm [23], peripheral neuropathy [24,25], cognitive impairment [26], tumor lysis syndrome [24], and organ damage (cardiotoxicity, hepatotoxicity, nephrotoxicity, and ototoxicity) [25] are the adverse effects reported with antineoplastic agents.

Drug resistance is the main cause of treatment failure in cancer patients, the key mechanism of which is the potential efflux of cancerous cells, as these usually produce high amounts of efflux pumps such as p-glycoprotein [27]. Another mechanism responsible for drug resistance is gene amplification, which may lead to defective apoptotic pathways or the restoration of proliferative ability of cancerous cells. Mutations in genes that produce target proteins(tubulin) may result in the prevention of drugs from binding to target proteins [28]. The pharmacokinetic variability of cancer fighting drugs between patients is another concern that is difficult to deal with, and which may put clinicians in the challenging situation of choosing the right dose for the right patient to achieve an optimal therapeutic response [29]. In a randomized clinical trial, 68% of patients with metastatic colorectal cancer were found to be underdosed and 17% were overdosed when treated with 5-fluorouracil [30].

The potential impact of vitamins and minerals on long term health, cognition, healthy development, bone mineralization, and aging has been supported by experimental, animal, and epidemiological studies. Billions of people are suffering from malnutrition and micronutrient deficiencies in low-income countries, though inadequate micronutrient status might also now be an issue in industrialized states [31]. Micronutrients have several health benefits, including bone formation, tissue maintenance, countering oxidative stress, serving as cofactors and coenzymes to certain enzymes, and the regulation and coordination of most bodily functions [32]. The success of treatment in the cancer recovery process may be highly influenced by the nutritional status of patients. An insufficient diet could possess detrimental effects on immune status, tolerance to treatment, and ability of chemotherapy to destroy cancerous cells. More importantly, mortality rates are almost 30% higher in malnourished cancer patients [33]. According to the European Society for Clinical Nutrition and Metabolism (ESPEN) guidelines, cancer patients who consume less than 60% of their daily energy requirements for 7–10 days cannot possibly have an adequate supply of micronutrients [33].

The objective of this manuscript is to review the literature to elaborate the possible link between micronutrients and cancer pathogenesis of GIT and hepatic origins. Additionally, the molecular targets of micronutrients in cancer therapeutics are also highlighted. Electronic databases including Google Scholar, PubMed, Scopus, and Web of Science were searched using the keywords “Cancer therapy” AND “Micronutrients” AND “Cancer” OR “Gastrointestinal cancer” OR “Hepatic cancer” AND “Molecular targets” OR “Antioxidant effects” OR “Apoptosis targeting” OR “Antiproliferative mechanisms” OR “Anti-angiogenic effects”.

## 2. Micronutrients and Cancer: What We Need to Know?

In recent decades, most cancer patients have been taking vitamins and minerals such as vitamin D and selenium, with the aim of improving the effectiveness of the standard therapy and/or to decrease the undesirable side effects of the treatment together with the underlying disease [34]. Data collected from 2003 to 2010 within an Intergroup Phase III Breast Cancer Chemotherapy trial (S0221) revealed that 48% of patients were taking multivitamins, 34% were taking only calcium, 20% were receiving vitamins C and D, and omega-3 fatty acids, and 15% were supplementing with Vitamins B6, B9 and E [35]. However, there are justifiable concerns from an oncological point of view that taking dietary supplements during the course of cancer therapy may affect the efficacy of chemotherapy [35,36,37,38]. Thus, micronutrients must be selected appropriately, and should be taken at the right time so as not to reduce the effectiveness of cytoreductive therapy. Improved patient compliance, decreased frequency of adverse events, and lowered rates of treatment discontinuation have been evidenced in recent studies where selected micronutrients (vitamin D and selenium) and L-carnitine, were added to concurrent anticancer medications. An improved response to the cancer therapies has been noted, which improves the prognosis and patient’s quality of life [39,40,41].

Cancer disease and therapy can influence the patient’s nutritional status, which may exert a critical impact on the course of the disease, the efficacy of therapy and the risk of disease and/or treatment induced complications including fatigue, depression, impaired immunocompetence, and delayed wound healing. That is why laboratory-validated supplementation of micronutrients to cancer patients is now an important aspect in the concept of adjuvant and complementary oncological treatment, and therefore, in addition to the energy substrates (carbohydrates, proteins, and fats), it is important to ensure the optimal supplementation of immunostabilizing micronutrients [33]. Supplies of immunomodulatory and antioxidant micronutrients (Vitamin D and selenium) and vitamins with low storage capacity (Vitamin B1, C, B9 and K) are particularly critical in such patients [42,43,44,45,46,47]. Elevated markers of oxidative stress due to inadequate supplementation of antioxidant micronutrients [48,49] and increased risk of bleeding in association with zinc deficiency in cancer patients with poor nutritional status, have been evidenced [50].

Research is now equally focused on micronutrient supplementation and anticancer therapy, as micronutrient deficiencies are negative regulators of the course of malignant disease and the antitumor efficacy of treatment. Numerous studies have highlighted the potential role of micronutrient supplementation in improving the quality of life and the prognosis of cancer patients. A cohort study recruiting 1129 patients with lung cancer conducted by Jatoi et al., demonstrated a reduction in mortality rate of 26% in patients taking micronutrient preparations, in comparison to those not receiving such supplements. The mean survival of micronutrient supplementing and non-supplementing patients was 4.3 and 2 years, respectively [51].

Supplementation of patients with ovarian cancer (who were treated with cyclophosphamide and cisplatin) with multiple antioxidants (selenium 200 µg, vitamin C 800 mg, vitamin E 144 mg, β-carotene 60 mg, vitamin B2 18 mg, and vitamin B3 180 mg) every day for three months showed a significant improvement in the immune status of the patients, with decreased frequency of chemotherapy-induced adverse events. A significantly higher level of selenium, enhanced erythrocyte peroxidase activity, and higher leukocyte (neutrophils and granulocytes) count were observed in patients taking regular antioxidant supplements. Moreover, the incidence of chemotherapy-induced side effects including nausea, vomiting, anorexia, flatulence, abdominal pain, stomatitis, alopecia, malaise, and weakness were considerably lower in antioxidant receiving patients, compared to controls. In addition, cisplatin-induced neurotoxic symptoms occurred in one patient in the antioxidant group and two patients in the control group [52].

The clinical use of antioxidant supplements during cancer therapy is, however, still controversial and not fully justified, as the tumor reductive effects of some cytostatic agents and radiotherapy partially involve the formation of free radicals [37], though the extended list of cytostatic drugs in clinical practice nowadays do not primarily act through oxidative stress, such as antimetabolites (methotrexate), nitrogen mustard derivatives (cyclophosphamide), platinum complexes (cisplatin), vinca alkaloids (vinorelbine), anthracyclines (epirubicin), and taxanes (paclitaxel) [33]. If antioxidant nutrients interfere with the efficacy of standard anticancer therapy, then patients should not be allowed to take antioxidant supplements [33]. In contrast, antioxidants such as retinoids, vitamin C, vitamin E, and selenium have other essential metabolic functions in addition to radical scavenging properties, i.e., immunomodulation, apoptosis induction, and regulatory effects on cell proliferation and differentiation. It is possible that they may halt tumor growth through enhanced cell differentiation and apoptosis [53,54].

As recommended by the American Institute for Cancer Research (AICR), cancer patients with active chemotherapy and radiotherapy should not take micronutrient supplements containing antioxidants in a daily dose greater than the corresponding tolerable upper intake level. According to the same recommendation, the vitamin and mineral supplements can generally be regarded as safe in these patients if the daily doses are in the range of the recommended daily allowance (RDA) [55,56,57]. Following AICR recommendations for cancer prevention, all attempts to fulfill the needs of essential nutrients are to be exercised through dietary sources, before considering vitamin and mineral supplementation. Cancer patients should only be advised to take micronutrient supplements in case of nutritional problems and/or significant weight loss, and only to cover the basic supply of essential micronutrients. This approach will prevent a high-dose micronutrient intake and will compensate for potential nutrient deficiencies.

## 3. A Possible Link of Micronutrients with GI and Hepatic Cancers

Increasing evidence has suggested the important role of dietary habits and nutrient intake in the prevention of gastric malignancies. A report published by AICR and the World Cancer Research Fund (WCRF) indicates a possible decrease in the risk of gastric cancer with high consumption of fruits and non-starchy vegetables [58]. Certain micronutrients such as vitamins C and E, zinc, and iron have proven efficacy against *H. pylori* infections, and thus can be helpful in modulating the immune response while decreasing the risk of carcinogenesis [59,60]. The scientific data support the great contribution of microbial species to the development of cancers attributable to infectious agents [61], and thus it is also possible that micronutrients may influence the composition of gut microbiota that would have direct effects on *H. pylori*-induced disease states [62].

Plasma and dietary levels have been analyzed for several micronutrients, leading to the identification of retinol, vitamin C, and selected carotenoids among dietary components that may explain the protective role of fruits and vegetables against gastric carcinogenesis [63,64,65]. An Italian case control study showed the favorable effects of vitamin E, α-carotene, and β-carotene, and subsequently the detrimental effects of sodium intake even at intermediate levels, on gastric cancer [66]. Another hospital-based study displayed an inverse relation between gastric cancer and the high consumption of antioxidant vitamins (C, E and B3), potassium and iron, as well as with a low intake of sodium [67].

A large population-based case-control study conducted by Sun and colleagues demonstrated a lower risk of colorectal cancer with a dietary and supplementary intake of vitamin D, C, B2, B9, and calcium. Conversely, iron intake was associated with a higher risk of the disease (which may be due to provoking a chronic inflammation secondary to iron overload) [68]. More importantly, after exclusion of supplement users, the anticancer potential of vitamin D and calcium (i.e., from dietary sources only) remained significant. In a recent retrospective cohort study on 315 peri- and post-menopausal women undergoing colorectal and osteoporosis screening, serum vitamin D levels correlated with the presence and histological grading of colorectal adenomas. A total of 77 colorectal lesions were identified in 66 patients. Vitamin D insufficiency (<30 ng/mL) and deficiency (<20 ng/mL) were recognized in 79.4% and 35.2% of patients, respectively [69]. On the other hand, it should not be overlooked that abdominal irradiation in patients with gastrointestinal cancer can lead to bone loss and osteoporosis, thus increasing the risk of fracture of the vertebrae exposed to rays. Therefore, in conjunction with therapeutic abdominal irradiation, the advisability of adopting a calcium and vitamin D supplementation should be considered and weighed both as a complementary anti-cancer therapy and for the prevention of osteoporosis and consequent bone fractures [70]. A large 17-cohort study involving 5706 colorectal cancer patients and 7107 control participants with a wide range of circulating 25(OH)D showed that higher circulating 25(OH)D was related to a significantly lower risk of colorectal cancer in women and to a lower (but not significantly so) risk in men, with an optimal 25(OH)D concentration for colorectal cancer risk reduction of 75–100 nmol/L [71].

Since the liver is extensively involved in the metabolism of a wide range of xenobiotics, it is thus more prone to oxidative damage. An adequate consumption of antioxidant nutrients could decrease the markers of oxidative stress, which would protect hepatocytes from associated injuries [72]. Hepatic disorders could possess a profound impact on the nutritional state of patients, including vitamins and minerals, and micronutrient deficiencies may impair metabolic processes at the biochemical and cellular level, and thus could further worsen the clinical situation of the patients even before physical and clinical alterations are seen. Evaluation of the micronutrient status in all patients with chronic and advanced liver diseases is an essential part of a comprehensive nutrition assessment, as optimization of the metabolic state is crucial to prevent disease progression to liver cirrhosis and HCC. Thus, early intervention with micronutrient supplementation could be a fruitful approach to improve clinical outcomes and patients’ quality of life [73,74]. Patients with hepatic disorders are usually predisposed to the development of hepatic cancer. Zinc and vitamin D deficiency, and elevated serum levels of copper are considered to be among the risk factors for HCC [75,76,77]. Most of the patients with HCC have underlying cirrhosis, and are inherently at increased risk for micronutrient deficiencies [78].

The role of individual micronutrients in countering GI and hepatic carcinogenesis is described below. Table 1 presented the selected micronutrients supported by their Recommended Dietary Allowance (RDA) levels and dietary sources.

### 3.1. Vitamin D

Vitamin D has a key role in regulating the level of serum phosphorus and calcium to maintain neuromuscular activities, homeostasis of bone through the regulation of calcium absorption for bone mineralization, and normal cellular functions [94]. Much is known about the classic role of vitamin D in bone metabolism, but this micronutrient appears to play a multifaceted spectrum of roles. Vitamin D status and incidence of cancer have an inverse association, as shown by epidemiological studies, suggesting that vitamin D deficiency could be one of the risk factors in carcinogenesis [80]. In 1980, it was proposed that vitamin D accounted for the high mortality rate from colorectal cancer in populations with less exposure to sunlight [95]. Vitamin D binds to and activates nuclear vitamin D receptors (VDR), causing transcriptional activation and repression of the target genes, and an association of the VDR *Bsm*I polymorphism with colorectal cancer has been observed [92]. It has been implicated in a number of anticancer activities such as anti-proliferation, differentiation and apoptosis induction, suppression of invasion and metastasis, anti-inflammation, and inhibition of angiogenesis [93]. In mice with colitis (a known risk factor for colorectal cancer), high consumption of vitamin D in the diet attenuated colon inflammation via downstream regulation of mitogen-activated protein kinase (MAPK) signaling, suggesting its possible role in inflammation-associated carcinogenesis [96]. While studying the impact of vitamin D on cellular metabolism in an experimental model of colorectal cancer, Zuoet al., noted a suppression of glycolysis via promoting c-Myc degradation through activation of long non-coding RNA *MEG3* [81]. The cellular metabolism is always altered in cancerous cells, characterized by glycolysis with lactate production and higher glucose uptake, and glycolysis is mainly driven by c-Myc in normoxic conditions, which upregulates glycolytic enzymes (lactate dehydrogenase A and hexokinase 2) [97,98,99,100].

Recent studies have elaborated on the interaction between the gut microbiome and immunity in colon cancers [101,102], and vitamin D has been reported to positively regulate the gut microbiota [103]. In a research model of dextran sodium sulfate-induced colitis, supplementation of mice with calcitriol showed fewer numbers of *Helicobacter* species and severity of colitis, as compared to the control group [104]. As measured vitamin D levels showed an inverse relation with colorectal cancer, the positive impact of vitamin D supplementation on the incidence of cancer and survival of patients with colorectal cancer remains conflicted. Nevertheless, a meta-analysis of prospective studies showed a low incidence of colorectal cancer in patients with an adequate intake of vitamin D [105]. A Cancer Prevention Study II Nutrition Cohort recruiting 1,111 participants (with a mean age of 73 years) at diagnosis of invasive, non-metastatic colorectal cancer found a decrease in all-cause and cancer-specific mortality with milk and calcium intake when evaluated after a mean follow-up of 7.6 years, but no association with vitamin D was found for either mortality outcomes [106].Apc1638N/+ mice supplemented with calcium and cholecalciferol showed a reversal of western diet-induced growth, promoting changes in the colonic epithelium [107].

As an intake of vitamin D can cause hypercalcemia effects, and thus its use for the prevention and/or treatment of cancer is not recommended by numerous studies, its analogs have yielded fewer hypercalcemic effects [108]. Paricalcitol, an analog of calcitriol, suppressed the growth of gastric cancer cells via regulation of the cell cycle, apoptosis, and inflammatory pathways without causing hypercalcemic effects [109,110,111]. A novel analog of cholecalciferol (1α-hydroxy-24-ethyl-cholecalciferol (1α[OH]D5)) has been shown to inhibit the growth of carcinogen-transformed MCF-12F breast epithelial cells and hormone-sensitive BT-474 breast cancer cells [112], and it should be investigated further in GI and hepatic cancers. A study showed that vitamin D acts through the hedgehog signaling pathway in gastric cancer cells, where it reduced cell viability by suppressing the expression of several hedgehog signaling target genes including patched1 and Gli1 [113].

Based on epidemiological studies, the association of vitamins with HCC is still unclear, but biochemical evidence clearly indicates the responsiveness of HCC cells to the repressive effects of vitamin D and its analogs in HCC patients with a mean age of 59.9 years [80]. Results of the EPIC trial demonstrated a lower risk of HCC with higher baseline 25-hydroxy vitamin D levels [75]; however, an interesting variation was noted among dietary sources i.e., vitamin D from nondairy sources, showing an inverse or null association with HCC risk while vitamin D from dairy sources displayed a higher risk for disease [114].

The primary causes for this increased risk of HCC with the increased consumption of dairy products are their potential to interact with insulin-like growth factor (IGF) pathway components, and the enhanced exposure to alfatoxins. The results of a research study demonstrated 16.8 μg/L higher IGF-1 concentrations with each 400 g increment in daily dairy intake, while each 200 g increment in daily milk was associated with 10.0 μg/L higher IGF-1 levels in subjects comprised both of men and women (*n* = 526) aged 18–80 years [115]. Conversely, no association was found between the intake of yoghurt or cheese and circulating IGF-1 concentrations. Alfatoxin is one of the more potent hepatocarcinogens present in milk. The European Food Safety Authority has shown a very low concentration of aflatoxin M1 (the hydroxylated metabolite of aflatoxin B1) measured in milk samples collected from different European countries [116]. However, the daily ingestion of aflatoxin M1 may remain significant, given that milk is consumed at a high rate across the Europe [117]. The variation in the association of milk, cheese, and yoghurt with enhanced HCC risk is possibly due to differences in the content of IGF-1 and aflatoxin, present in the dairy products.

Vitamin D possesses anti-fibrotic, anti-proliferative, and immunomodulatory actions in many cancer cells that express VDR, including HCC cells [118]. Calcitriol exhibited growth inhibitory effects on HCC cell lines (4 human and 1 rat HCC cell line), with the highest efficacy in two human cell lines comprising of HepG2 and Hep3B [119]. Vascular endothelial growth factor (VEGF) and epidermal growth factor receptor are also vitamin D targeting signaling pathways, implicated in the protection against HCC [80]. Vitamin D is capable of decreasing epidermal growth factor receptor expression, resulting in the inhibition of MAPK and subsequent cellular differentiation, apoptosis, and growth inhibition [120]. Similarly, through inhibition of endothelial cell proliferation and hence angiogenesis, vitamin D may prevent VEGF-mediated hepatocarcinogenesis via inhibition of blood vessel formation [121,122].

Vitamin D imparts immunomodulatory properties via acting on a variety of immune cell types. Classically, vitamin D activates the innate immune system (useful in fighting effectively against bacterial infections) and downregulates the adaptive immune system resulting in repressing the self-reactivity of immune cells [123]. Inflammation is one of the essential hallmarks of colorectal cancer, as increased risk of cancer has been seen in patients with inflammatory bowel disorders such as ulcerative colitis and Crohn’s disease, and it is highly probable that vitamin D may alter cancer pathogenesis through the regulation of immune cells [124]. Vitamin D supplementation in patients with colorectal adenoma decreased the inflammation scores as calculated from the plasma levels of anti-inflammatory mediator (IL-10) and pro-inflammatory markers (C-reactive protein, TNF-α, IL-6, IL-1β, and IL-8) [125]. Interestingly, deletion of the myeloid cell-specific or non-intestinal epithelial cell-specific VDR resulted in the aggravation of clinical symptoms in an experimental mouse model of colitis, with increased expression of pro-inflammatory cytokines in the colon [126]. Conversely, supplementation of healthy subjects with low vitamin D western diets and calcitriol showed an induced expression of genes involved inflammation and immune responses in healthy subjects, suggesting that calcitriol may induce adaptive immune responses [127]. This shows the complexity in the relationship between vitamin D status and the immune system, probably depending upon the dose and nature of the VDR agonists (cholecalciferol and calcitriol). Another beneficial mechanism of calcitriol in colorectal cancer is the potentiation of antibody-dependent cell cytotoxicity in monoclonal antibody-receiving (epidermal growth factor receptor inhibitors or vascular endothelial growth factor inhibitors)patients [128].

Serum levels of calcidiol (being an appropriate parameter for serum indication of vitamin D) below 20 ng/mL are termed as vitamin D deficiency, while 30 ng/mL are considered to be vitamin D insufficiency. Patients with severe vitamin D deficiency, i.e., a calcidiol serum level below 10 ng/mL, are thought to be more prone to hepatic carcinomas. According to the Endocrine Society Guidelines (ESG), the serum level of vitamin D in hepatic cancer patients must be greater than 30 ng/mL. The ESG recommended a daily dose of 6000 IU vitamin D for up to 8 weeks or 50,000 IU per week until the serum level of vitamin D exceeds 30 ng/mL [129,130,131].

The above findings have indicated vitamin D as one of the potential nutraceuticals with chemopreventive effects (particularly in colorectal cancers and HCC) as it regulates several signaling pathways along with modulating the gut microbiota. However, extra care should be exercised in patients that are at high risk for hypercalcemia. In addition, higher levels of vitamin D intake could be achieved from the consumption of non-dairy sources rather than dairy sources, with the aim of preventing hepatic carcinogenesis.

### 3.2. Antioxidant Vitamins

Oxidative damage to DNA is one of the crucial steps in carcinogenesis, which may arise as a consequence of exposure to xenobiotics or may be due to endogenous production of oxidizing compounds. Numerous studies have explored the effects of the ingestion of antioxidant compounds (vitamin A, C, and E) on oxidative damage to DNA and the risk of cancer [132]. However, the use of antioxidants aimed at preventing cancer risk is currently the subject of heated debate due to the lack of solid research findings, as some studies suggest that the use of antioxidants in patients with cancer can decrease the risk of carcinogenesis with amelioration of toxic effects of the therapy, while others suggest that it may interfere with the efficacy of chemotherapy and radiotherapy [133].

#### 3.2.1. Vitamin A

Larsson et al., evaluated the effects of vitamin A, retinol, and carotenoids against the risk of gastric cancer in adult subjects (aged 45–83 years) in a prospective cohort study [65]. After an average follow-up of 7.2 years, it was concluded that high consumption of dietary vitamin A, retinol, α-carotene, and β-carotene, and intake of vitamin A, and retinol from combined dietary and supplemental sources were significantly associated with a lower risk of gastric cancer. The impact of vitamin A deficiency on mucosal immunity has been widely observed, possibly through an enhanced T-helper type 1 (Th1) response and increased pro-inflammatory cytokine levels, resulting in upregulating the inflammatory cascade [134]. In rats, downregulation of the retinoic acid receptor (RAR)–α-mRNA, enhanced dendritic cells, and increased IL-12 secretion in the intestinal mucosa were noted [135]. The influence of vitamin A deficiency on colitis and the development of colorectal cancer was assayed in mice by Okayasu et al., [136]. Colitis was more severe in vitamin A-deficient mice, compared to vitamin A-supplemented mice. Moreover, vitamin A-deficient mice were found to have reduced colonic subepithelial myofibroblasts and ratio of IgA^+^/IgG^+^ cells, with enhanced CD11c^+^ dendritic cells, and subsequently higher rates of colorectal carcinoma with colitis following azoxymethane injection. The decrease in vitamin A concentration in subepithelial myofibroblasts in vitamin A-deficient mice was evocative of alterations in colonic crypt niche function.

A few epidemiological studies have found higher pre-diagnostic serum levels of retinol with decreased HCC risk [137,138], although no or even negative associations have been reported in some studies. No significant correlation was found in two Chinese cohort studies for dietary vitamin A [82] or β-carotene supplementation in an alpha-tocopherol, beta-carotene (ATBC) Cancer Prevention Study [139]. A well-established case-control study demonstrated a significant decrease in HCC risk in newly diagnosed primary liver cancer cases for patients aged 18–80 years, with greater intake of total vitamin A, retinol, and carotenes in amounts of 1000 µg retinol equivalent per day or greater from dietary sources [140]. Retinoids exert their pleiotropic effects by selective, high-affinity binding to nuclear retinoid receptors, where growth suppression and induction of differentiation are canonical mechanisms. The binding of ligands to nuclear retinoid receptors orchestrate the preventive and therapeutic actions of these agents through positive and negative regulation of certain genes [141,142,143,144]. Non-genomic targets of retinoids that may be helpful in cancer prevention include suppression of kinase cascades such as phosphoinositide 3-kinase (PI3K) and nuclear factor kappa-light-chain-enhancer of activated B cells (NFκB) [145].

#### 3.2.2. Vitamin E

The association of serum vitamin E levels was evaluated with upper GI cancers in a case-cohort design by Taylor and colleagues in adult patients, aged 57 to 60 [83]. Serum levels of α-tocopherol and γ-tocopherol were measured in patients with incident esophageal squamous cell carcinoma, gastric cardia cancer, or gastric non-cardia cancer. Results analysis only revealed the preventive role of α-tocopherol (serum level measured was 140 µg/dL) in upper GI cancers, as no connection between γ-tocopherol (serum level measured was 15.4 µg/dL) and the development of any of these cancers was found. In the National Institutes of Health-AARP (NIH-AARP) Diet and Health Study, Carman et al., observed little evidence of possible links between dietary α- and γ-tocopherols with the risk of esophageal and gastric cancers, with the exception of the positive correlation of α-tocopherol with gastric non-cardia adenocarcinoma. Similarly, results were mainly null for supplemented vitamin E, except for an inverse association of high doses of vitamin E with gastric non-cardia adenocarcinoma [146].In vitro and in vivo studies depicted the inhibitory effects of vitamin E succinate on colon cancer and tumor metastasis of the liver via promotion of tumor apoptosis and suppression of cell proliferation [84]. No positive impact had previously been found for dietary tocopherols with colon cancer in a case-controlled study, but most importantly this found that the risk of carcinogenesis was slightly increased with γ-tocopherol [147].

A report from two cohort studies conducted in China revealed a reduction in the risk of hepatic cancer with vitamin E intake from food and supplemental sources, unlike other supplements (vitamin A, B, C, multivitamin, or calcium) which were found to be unrelated to liver cancer risk [82]. No considerable difference was found between dietary and supplemental vitamin E. In a randomized, double-blind, placebo-controlled trial, α-tocopherol was noticed to have a positive impact on mortality rate related to chronic liver disease in male patients aged 50 to 69, but no effect was observed on hepatic carcinomas, while higher concentrations of β-carotene and retinol possessed therapeutic benefits with regards to chronic liver disease mortality and liver cancer [148]. In an ATBS study, supplemental intake of α-tocopherol, β-carotene, or both, did not reduce the risk of chronic liver disease and liver cancer relative to placebo, neither during the intervention nor in post-intervention periods in male smokers aged 50 to 69 years [139].

The possible anticancer mechanisms of action for tocopherols have been thought to be anti-oxidative effects by trapping of reactive nitrogen species, COX-2 inhibition, upstream regulation of Peroxisome proliferator-activated receptor gamma (PPAR-γ) nuclear receptors, inhibition of cell growth, and apoptosis induction [149,150,151]. Importantly, the main reason proposed for the non-satisfactory clinical efficacy of tocopherol isoforms in cancer patients is their low bioavailability, due to the saturation of transporter proteins (mainly SR-B1 and NPC1L1) at high concentrations affecting their systematic absorption [152]. Different delivery systems could be used to improve the bioavailability of tocopherols and to enhance their effectiveness in cancer patients [152].

#### 3.2.3. Vitamin C

Attributable to its strong antioxidant potential, a high intake of vitamin C may serve a protective role in GI cancer, especially in upper GI malignancies [153]. In a randomized controlled trial, patients (mean age: 51.1 years; 46.1% male) with confirmed histologic diagnoses of multifocal non-metaplastic atrophy and/or intestinal metaplasia were assigned to treatment with *H. pylori* triple therapy and/or dietary supplementation of vitamin C and β-carotene, or corresponding placebos [85]. In this high-risk population study, effective treatment with *H. pylori* standard therapy and dietary supplementation with antioxidant micronutrients significantly interfered with the precancerous process, mainly by enhancing the rate of regression of cancer precursor lesions. On the other hand, supplementation with antioxidant micronutrients (vitamin C, E, and β-carotene) was not found to be an effective tool for gastric cancer control in a high-risk population [154]. Patients considered at high-risk for gastric cancer were supplemented with vitamin C (750 mg/day), vitamin E (600 mg/day), and β-carotene (18 mg/day), or placebo, but the effects of nutrients on precancerous lesions were not significant, as the regression rates were 116.5 and 109.4 in the vitamin and placebo-treated group, respectively. Jacobs et al., also did not find any substantial effects of vitamin C or E supplements on overall colorectal cancer mortality in a large American Cancer Society cohort of male and female patients [155].

In CCL4-challenged mice, vitamin C offered hepatoprotection against hepatic lesions through attenuation of inflammatory stress [156]. The antioxidant markers were restored in mouse liver, with a reduction in the number of TNF-α positive cells and downregulation of the intrahepatic expression of toll-like receptor-4 (TLR-4) mRNA (a vital regulator of inflammation). In human HCC cell lines and HCC patient-derived xenograft models, vitamin C induced cell death in liver cancer cells [86]. Vitamin C uptake via sodium-dependent vitamin C transporter 2 (SVCT-2) resulted in increased intracellular oxidative stress, and subsequently DNA damage and ATP depletion, resulting in cell cycle arrest and apoptosis [86].

In vivo studies using animal models have supported the possible link between increased levels of exogenous antioxidants (β-carotene, vitamin A, vitamin E, and vitamin C) and prevention against free radical-induced damage, associated with carcinogenesis. However, the evidence from human studies to support the usage of antioxidant vitamins as potential chemopreventive agents is very weak, as these studies yielded mixed results. Many observational studies have shown an inverse relationship between antioxidant vitamins and development risk for cancer, but the results obtained from observational studies must be viewed with caution because of inadequate control for biases, that may influence study outcomes. On the other hand, randomized clinical trials did not support the chemopreventive effects of antioxidant vitamins. In light of the available literature, it is wise to encourage the increased intake of fruits and vegetables as one of the dietary approaches to decrease the risk of developing cancers rather than antioxidant vitamin supplements.

### 3.3. Zinc

Zinc is a promising chemopreventive dietary agent, as it is integral to many transcription factors and proteins regulating key cellular and biochemical functions, such as cellular response to oxidative stress, DNA replication, DNA damage repair, cell cycle progression, and apoptosis [157]. Zinc supplementation could decrease angiogenesis and the presence of inflammatory cytokines, with apoptosis induction in cancer cells [158]. The human body cannot store zinc, and an improper diet can rapidly lead to zinc deficiency, while many epidemiological studies have confirmed a link between zinc deficiency and an increased risk for cancer development [159]. Studies have advocated the normalization effects of zinc on the histoarchitecture and antioxidant status in the colon of rats during the initiation and promotion phases of experimentally induced colon carcinogenesis [88,160]. An in vivo study showed a membrane-stabilizing effect by restoration of membrane fluidity and surface changes in rats, following 1,2 dimethylhydrazine (DMH)-induced colon carcinogenesis [161]. Nutritional deficiency of zinc in rats increased esophageal cell proliferation and the incidence of chemical-induced esophageal tumors. Replenishing zinc with a zinc sufficient diet reduced these effects by rapid induction of apoptosis in esophageal epithelial cells [89]. In another study, esophageal cell proliferation was effectively reversed with zinc replenishment, and tumor incidence was reduced from 100% to 14% in pair-fed zinc-replenished rats and 26% in rats with zinc-sufficient diet *ad libitum* [162].

A report by Jaiswal and Narayan suggested the stabilization of the levels of wild-type adenomatous polyposis coli at the post-transcriptional level with zinc treatment, causing growth arrest in colon cancer cells [163]. Serum zinc levels were clinically correlated with GI cancer, and it was found that serum zinc levels are more closely related to advanced gastric cancers [90]. A randomized clinical trial on patients with colorectal cancer undergoing chemotherapy with male to female ratio of 4:6 for zinc group (mean age: 62.5 years) and 5:9 for the placebo group (mean age: 63.8 years) demonstrated increased superoxide dismutase (SOD) activity and maintained vitamin E concentration with zinc supplementation during chemotherapy cycles, however, no effects were seen on oxidative stress markers [164]. Induction of SOD activity indicated the production of stable free radicals. A randomized clinical trial has been currently registered on clinicaltrials.gov (accessed on 12 February 2021) (NCT03819088) to evaluate the effectiveness of zinc supplementation in patients with GI cancers (gastric carcinoma, liver and intrahepatic bile duct carcinoma, unresectable esophageal carcinoma, and unresectable pancreatic carcinoma) that cannot be removed by surgery and are currently receiving chemotherapy. Moreover, the reciprocity in effects of zinc on *H. pylori* pathogenesis likely underpins the differences observed in gastric cancer risk [62].

The effects of oral zinc supplementation (600 mg/day for 7 days) on hepatic encephalopathy in patients with cirrhosis (with a mean age of 52.1 years for the zinc group and 52.7 years for the placebo group) was first reported by Reding et al., in 1984 [165]. Studies have shown significantly low zinc levels (≥55% decrease) in hepatic tissues (whether cirrhotic or non-cirrhotic) of HCC patients [91]. Stepien et al., reported a strong inverse association between serum zinc levels and the risk of hepatobiliary cancers in Europeans in multivariable models in HCC patients (with a mean age of 60.3 years and with 73% male subjects) [166]. Tamai et al., observed a correlation between increasing copper (Cu) and decreasing Zn levels with the progression of liver disease, and suggested the Cu/Zn ratio as a predictive marker for survival in HCC patients (139 men, 36 women, and mean age 71.1 years) [167]. A study with the same results was reported earlier by the Guangdong Liver Cancer Cohort [168]. Zinc can alter the immune responses in inflammatory liver pathologies such as liver cirrhosis, and thus can influence the clinical outcome of diseases including ascites, hepatic encephalopathy, and HCC [76]. Long-term supplementation of patients with zinc improved liver function as well as decreased the risk of HCC development [169]. The cumulative rate of HCC development, liver failure and mortality at 3 years was 9.5% in a zinc-treated group compared to 24.9% in the control group. A novel solid dispersion formulation of Zn-curcumin inhibited the growth of HCC, and also sensitized the effects of chemotherapy drugs in vitro and in vivo [170]. Administration of zinc-curcumin formulation alone or in combination with doxorubicin significantly reversed zinc dyshomeostasis, gut microbiota dysbiosis and intestinal mucus barrier disruption in HCC-bearing animals [170].

Zinc showed promising effects against GI cancers (upper and lower GI) and HCC involving both in vitro and in vivo studies. Oxidative stress is known to activate inflammatory pathways which may result in cancer initiation/progression and chemoresistance, whereas zinc possesses the potential to decrease free radicals (thus reducing oxidative stress) and to alter the immune response, which may help in mitigating carcinogenesis and sensitizing chemotherapeutic response towards conventional anticancer therapies. Optimal levels of zinc in the body may also help in reducing risk factors for cancer development, but this requires further study.

### 3.4. Selenium

The chemopreventive role of selenium is still debatable based on the available literature, as some studies have shown beneficial effects, but others have revealed no or even negative associations. Recently, a systematic review of randomized controlled trials and longitudinal observational studies found an increased risk of some cancer types in patients with selenium supplementation [171]. Fischer et al., sought to define the genetic basis for the observed selectivity of selenium, and they pointed out a p53-dependent DNA-repair response with seleno-L-methionine, which thus turns out to be protective against subsequent challenges by DNA-damaging agents [172]. Previously, the induction of p53 mediated cell cycle arrest and apoptosis had been observed with seleno-L-methionine in human colon cancer cell lines [173]. The potentiation of efficacy and selectivity of chemotherapeutic agents in nude mice bearing human tumor xenografts of colon carcinoma and squamous cell carcinoma of the head and neck has been reported [174], which may also improve the safety profile of anticancer drugs if found effective clinically.

Animal and clinical studies have highlighted the importance of adequate selenium intake in the protection against hepatocytes and reduction of the risk of primary hepatic cancers [175]. As hepatocytes are more susceptible to damage ensuing from oxidative, inflammatory, hypoxic, and endoplasmic reticulum stresses, and notably the expression of hepatic selenoproteins could be altered by these *noxae*, this suggests an interrelated regulation of cancer risk and prevention [175,176]. An accumulation of lipid and soluble peroxides and a dysregulation of hepatocellular function and differentiation may occur in individuals with low selenium intake and subsequently reduced selenoprotein expression [177]. The findings from a large prospective cohort provide evidence that suboptimal selenium status may be inversely linked with a risk of HCC development in Europeans [178]. Interestingly, selenium supplementation seems not to offer protection against carcinogenesis in populations with adequate selenium intake, i.e., North America, and the striking difference for this phenomenon lies in endogenous mechanisms, such as biosynthesis rates of the selenoprotein plateau once a sufficiently high intake is reached [175].

The chemopreventive role of selenium remains controversial, although the potential effects of selenium with regard to hepatic cancers have been reported in numerous studies. Selenium is an essential component of several enzymes and proteins (known as selenoproteins) that may aid in making DNA in addition to offering protection against cell damage. Selenium supplementation is not an effective agent for chemoprevention in subjects with adequate selenium consumption, as opposed to the selenium-deficient population.

## 4. Molecular Mechanisms of Micronutrients

Micronutrient intake is linked to a reduced risk of disorders such as cardiovascular disease, cancer, neuronal dysfunction, and cataracts [179]. An individual who consumes high amounts of vitamin C, barriers, citrus fruits, and tomatoes has a decreased incidence of proliferation and mutation [180]. Micronutrients exert anti-cancer action by the following underlying molecular mechanisms.

### 4.1. Antioxidant Effects

Vegetables and fruits are reported as promising antioxidant agents due to their micronutrients [181,182,183,184]. Epidemiological investigations find it challenging to delineate the effects of dietary antioxidants from the impact of many essential vitamins and dietary ingredients, although scientific proof of the nutritional benefits of ascorbic acid and vitamin D as antioxidants is emerging. Hydroxyl radicals, hydrogen peroxides, and superoxides are oxidative metabolites produced by metabolic pathways and radiation. Inappropriate intake of dietary antioxidants such as vitamin E and vitamin C can exacerbate radiation exposure [185,186,187]. Oxidative stress damages DNA and genetic material, which contributes to the development of aging and degenerative disorders including cancer [188]. With the progression of aging, oxidative lesions pile up in proteins and DNA [189]. DNA is oxidized by metabolites such as aldehyde and malondialdehyde generated from the metabolism of lipids. These metabolites assemble in proteins and DNA with developing age. The release of oxidants by phagocytic cells, as well as the resulting inflammation, is a potential provenance of NO that ultimately leads to cardiac disorders and carcinomas [190]. These free radicals contribute to the genetic mutation of DNA which leads to neoplasm and necrotic damage to cells [191].

The antioxidant micronutrients reported in vegetables have curative effects against GI and lung cancers [182,192]. The administration of micronutrients including beta-carotene, vitamin E, and vitamin C in patients suffering from oxidative damage to DNA has led to significant recovery. In China, a clinical study on cancer progression has reported that the supplementation of beta-carotene, selenium, and vitamin E markedly improved the condition of cancer patients [193,194]. Since gamma-tocopherol is a strong nucleophile, it binds electrophilic mutagens that reach the cell membrane. Glutathione is a strong nucleophile and antioxidant present in the soluble portion of the cell. Alpha-tocopherol and gamma-tocopherol act as a nucleophile and an antioxidant in the cell membrane, respectively. Alpha-tocopherol neutralizes electrophile mutagens such as NO. Alpha-tocopherol combines with NO and forms nitro–alpha-tocopherol complexes which recover the oxidative damage to proteins and DNA in cancer patients [195,196].

**Figure 1:**
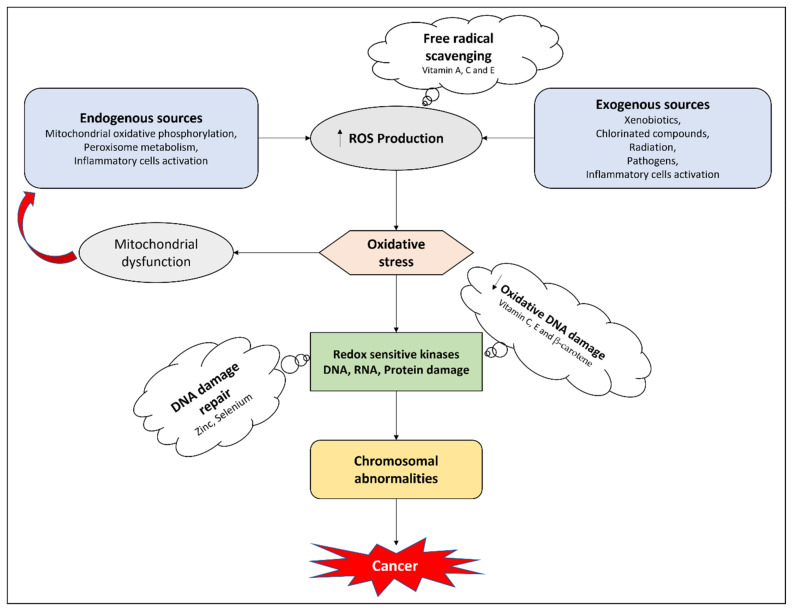
Potential mechanisms of micronutrients in countering oxidative stress. Increase (↑); decrease (↓).

Vitamin D supports the antioxidant response via activating cellular signals which are responsible for the reduction of thioredoxin. Vitamin D also induces the expression of superoxide dismutase as well as downregulating levels of glutathione via elevating the levels of glucose-6-phosphate dehydrogenase [197]. Vitamin C, a potent antioxidant, is reported to be a potential replenisher of other antioxidants such as vitamin E throughout the body [198]. Ascorbic acid decreases free radicals, unstable nitrogen, oxygen, sulfur, and hydrogen atoms. Studies have shown that vitamin C preserves plasma lipids from peroxidative devastation caused by aqueous peroxyl radicals in human plasma [199]. In both in vivo and in vitro studies, higher doses of vitamin C have shown anticancer activity on tumor cells, thus presenting vitamin C as a pro-oxidative drug that inhibits hydrogen peroxide development in tissues, rather than merely serving as a radical scavenger [200,201]. Ascorbic acid is being studied to see whether, via reducing the harmful effects of free radicals through its antioxidant activity, it might hopefully avoid or postpone the onset of some chronic disorders such as cancer, wherein oxidative damage is a major factor. Vitamin E and vitamin C, the most important lipid-soluble antioxidants, may be effective. Vitamin C inhibits the synthesis of nitrosamines (carcinogens) via the reduction of nitrates [202,203]. Figure 1 highlights the potential antioxidant mechanisms of micronutrients.

### 4.2. Apoptosis Targeting

Different selenium species such as selenocysteine (SC), selenite (SeO_3_^−2^), and selenium dioxide (SeO_2_) trigger apoptosis in cancer cells by causing morphological and phenotypic modifications that are characteristic of apoptosis. The apoptotic molecular mechanism induced by selenium remains unclear. However, the apoptotic pathway induced by selenium is modulated by the synthesis of reactive oxygen species (ROS) [204,205]. ROS regulate the p53 activation pathway [206,207].Selenocysteine is involved in the initiation of the p53 pathway via protein phosphorylation of serine 15, 20, and 392 regions of p53. Moreover, upregulation of ROS is essential as the glutathione treatment decreases the phosphorylating process of p53 and thus prevents the apoptosis activated by SC. The mitochondrial dysfunction is activated by SC through segmentation of mitochondria, leading to loss of membrane potential which in turn results in the activation of the p53 pathway [208]. The activation of p53 upregulates the expression of Bcl-2−associated death promoter protein (BADP), tumor suppressor protein phosphatase and tensin homolog (PTEN), and Bcl-2−associated x gene (BAX) [209,210].

Researchers have reported that selenium causes the downregulation of B-cell lymphoma-extra-large and B-cell lymphoma-2 cells (anti-apoptotic proteins). The supplementation of selenium markedly elevated the mRNA levels of PTEN and p53 [204]. The elevated levels of BAX, BADP and the discharge of apoptotic mitochondrial proteins such as cytochrome C cause the aggregation of apoptosome, which leads to the activation of caspase-9 [209]. Caspase-9 provides the initiation step in the activation of caspase-3, 6 and 7 (downstream executioners). The downstreaming of the caspase cascade causes the cleavage of PRAP [211,212]. Researchers have established that selenium stimulates caspase-3,7 and 9 and in turn cleaves the PARP. The mRNA levels of caspase-3 and 9 are also found to be elevated with the supplementation of SeO_3_ [208].

Vitamin D is reported to have an apoptosis targeting effect via the regulation of anti-apoptotic mediators, suppressing BCL, BCL-XL and overexpressing BADP, BAX [120]. Calciferol has been reported in an in vitro assay to activate the p53 signaling pathway. Researchers have also established that vitamin D is involved in the elevation of BCl-2 genes (a pro-apoptotic protein). Apoptosis was induced by vitamin D administration in rat glioma cells via the upregulation of p53 and fragmentation of DNA [213].

**Figure 2:**
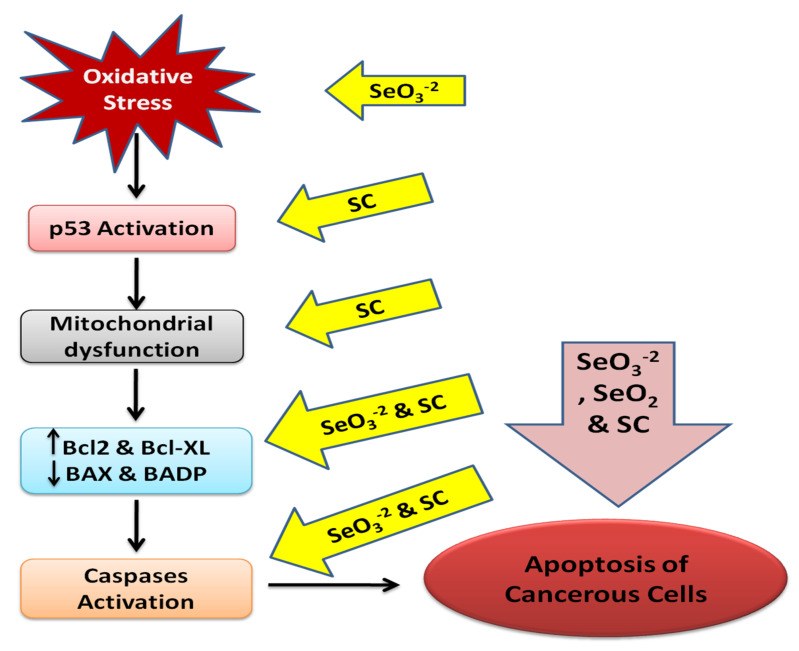
Selenium and derived compounds as a model for apoptosis targeting in cancer. Selenocysteine (SC), selenite (SeO_3_^−2^), and selenium dioxide (SeO_2_) act on different pathways of the apoptosis of cancerous cells via activating p53, caspase-9, Bcl 2, and inhibiting Bcl-2−associated x gene (BAX). Increase (↑); decrease (↓).

Selenium and derived compounds are presented as a model for apoptosis targeting in cancer in Figure 2.

### 4.3. Anti-Proliferative Mechanisms

Oncogenesis progresses through the three main steps of initiation, promotion, and proliferation. In the initiation step, cells at the tumor site develop somatic mutations that are transferred via ensuing mitosis cycles, resulting in progeny with gene expression defects that affect cell proliferation [214]. Promotion and proliferation occur due to the deregulation of cells, genetic abnormalities, and mutagenic promotion by further carcinogen exposure. Metastasis of cancerous cells leads to malignancy [215].

The pro-differentiating and anti-proliferating effects of micronutrients such as vitamin D have a prominent role in the prevention of malignancies [216,217]. Cell cycle modulation is regulated by an intricate system of connected regulators that instigate cellular proliferation. This regulation is controlled by cyclin proteins and their associated enzymes (cyclin-dependent kinases and cyclin-dependent kinase inhibitors). Malignancy takes place if the proliferation of neoplastic cells exceeds cell apoptosis (the molecular and biochemical signaling pathway for controlled cell death). The fact that several cancer drugs cause tumor regression by activating apoptosis highlights the importance of apoptosis in cancer-based scientific research [218]. The anti-proliferative function of vitamin D in malignant cells is based on cell-cycle disruption. Vitamin D inhibits cell growth by repressing a variety of important molecules concerned with cell cycle regulation. Treatment of MCF-7 cell lines in human breast cancer with vitamin D was found to suppress c-Myc, a recognized proto-oncogene involved in cell cycle regulation [219,220]. Vitamin D may thus enhance the activity of oncogene antagonists by suppressing the expression of certain oncogenes [221,222]. The G1 phase of the cell cycle was interrupted in ovarian carcinoma cells when supplemented with vitamin D via downregulating the cyclin-dependent kinase 2/cyclin E [223]. Vitamin D supplementation arrested the G0 and G1 phases of the cell cycle via inhibiting cyclin-dependent kinase 2 activity [224].

### 4.4. Anti-Angiogenic Effects

Angiogenesis is the synthesis of blood vessels in cancerous and normal cells. Endothelial cell proliferation and blood vessel development are hallmarks of pathological angiogenesis. Angiogenesis plays a key role in the metastasis and invasion of the neoplasm. Furthermore, circulating endothelial progenitor cells play a function in the formation of the blood vessels, and bone marrow-derived endothelial cell proliferation is connected to a number of tumors [225]. Since the expanding tumor mass requires an increased oxygen supply, neo-angiogenesis is a requirement for cancer progression in the tumor microenvironment. Hypoxia is frequently induced by tumor development, which facilitates hypoxia-inducible factor-1-dependent angiogenesis which is crucial for cancer development [226]. In many cancer cell lines, vitamin D has shown a suppressive effect on neo-angiogenesis. When vitamin D was added to an androgen-insensitive prostate cancer cell line, their proliferation was markedly reduced in hypoxic and normoxia conditions (similar to the ones in tumor cells). In breast cancer cell lines, vitamin D has shown inhibitory effects on vascular endothelial growth factor secretion. Moreover, it was reported that vitamin D inhibits angiogenesis via downregulating glucose transporter-1 and endothelin-1 which are necessary for neo-angiogenesis. HIF-1 translation and transcription are substantially downregulated in this molecular pathway [227].

Peyman and his coauthors established that excessive doses of ascorbic acid are involved in cancer prevention via suppressing the synthesis of blood vessels [228]. Mikirova et al., reported that a high dose of vitamin C modifies the metabolic activity of endothelial progenitor cells via lowering the ATPs level, thus inhibiting the proliferation of endothelial cells and the synthesis of blood vessels. Moreover, they established that a high dose of ascorbic acid prevents the synthesis of nitric oxide, which is a key agent in cancerous angiogenesis. Flavin adenine dinucleotide (FAD) and nicotinamide adenine dinucleotide phosphate are involved in the synthesis of nitric oxide. Thus, it is assumed that higher doses of vitamins alter the oxidation–reduction environment of the cancerous cells and thus nitric oxide synthesis is reduced by the production of peroxynitrite [229].

## 5. Micronutrients and Cancer Therapy Related Side Effects

Cancer patients usually face detrimental adverse events when undergoing chemotherapy and radiotherapy, which decrease the quality of life of patients in several ways [230,231]. Prescribing high doses of chemotherapeutic drugs to achieve an optimal anticancer response is becoming a common clinical practice nowadays, but this further increases the occurrence of side effects. Innovative methods of drug delivery, patient monitoring, and sophisticated support services enable clinicians to manage cancer patients with a range of therapy-induced side effects, but there remains a need for new agents or old molecules with novel indications that decrease the incidence of these effects while increasing the targeting potential of chemotherapeutics [232]. The alteration of the nutritional status for one reason or another in cancer patients with chemotherapy or radiotherapy was reported by Donaldson and Lenon in 1979 [233]. Thus, it was believed that the majority of the side effects may be related to nutritional deficiencies rather than the direct effects of anticancer drugs. The use of dietary supplements for such applications remains controversial, though some studies have shown promising results [56].

Antioxidant micronutrients may temper oxidative stress-related side effects of cancer therapy if administered in adequate amounts. The side effects most likely to be improved with antioxidant supplements include GI toxicities, mutagenesis, doxorubicin-induced cardiotoxicity, bleomycin-induced pulmonary fibrosis, and cisplatin-induced nephrotoxicity [234]. Antioxidants do not offer protection against certain side effects, such as myelosuppression and alopecia, and in cases where antioxidant compounds interfere with these effects it is most likely that they will also interfere with anticancer effectiveness [234]. A review of the 280 peer-reviewed in vitro and in vivo studies (including clinical trials) revealed that a sufficient intake of antioxidants and other nutrients does not interfere with the therapeutic modalities for cancer, but can most probably enhance the killing efficacy of these drugs, decreasing the occurrence of side effects, and protecting normal cells [235].

A randomized clinical trial found significant correlations between vitamin C supplementation, oxidative stress markers, and cisplatin-induced nephrotoxicity and ototoxicity [236]. In a six-month observational study, children undergoing treatment for acute lymphoblastic leukemia and with an inadequate intake of antioxidants (vitamin A, E, and carotenoids) were faced with increased chemotherapy-related adverse effects [237]. In his study, Nicolson claimed that antioxidant supplementation could decrease chemotherapy-induced side effects through the restoration of mitochondrial function, but should not be taken at the same time of day as the therapy [238].A meta-analysis of randomized clinical trials led to insufficient evidence for the alleviation of chemotherapy- and radiotherapy-induced side effects or improvement of the after-effects of surgery with selenium monotherapy [239].

## 6. Conclusions

The nutritional status and dietary factors are correlated with the prognosis of cancer disease, the efficacy of anticancer therapy, and associated side effects. Micronutrients from dietary and non-dietary sources have been found to be inversely related to the risk of GI and hepatic cancers. They constitute a valuable paradigm for cancer clinics in preventing the risk of cancer development and progression. Moreover, it is essential to optimize the nutritional status of cancer patients and to supplement them with adequate amounts of micronutrients to avoid malnutrition which can worsen the clinical situation of the patient. On the other hand, they may reduce the effectiveness of ongoing anticancer therapy, and thus it is recommended to fulfill micronutrient needs from dietary sources as a first priority, before relying on supplementation and to not exceed standard RDA limits set by guidelines. A limited number of studies also support a reduction in the occurrence of cancer therapy-induced adverse effects with micronutrients, especially antioxidant agents, which may also improve the quality of life of cancer patients. The current data available is too scarce to draw any final conclusion from the clinical point of view. More clinical studies (particularly randomized clinical trials) in large populations are needed to further evaluate the effectiveness of micronutrients in cancer patients and their subsequent benefits in reducing therapy-related side effects.

## Figures and Tables

**Table 1:** Selected micronutrients associated with GI and hepatic cancers.

Micronutrients	Underlined Cancer Types	RDA	Dietary Sources	References
Vitamin D	Colorectal cancer, HCC	15 µg	Egg yolks, tuna, salmon, sardines, mushrooms, cow’s milk, soy milk, orange juice, and fortified foods.	[32,79,80,81]
Vitamin A	Gastric cancer, Colorectal cancer, HCC	900 µg	Liver (animals and fishes) and egg yolk. Provitamin A carotenoids obtained from plant sources including deep green, yellow and orange fruits and vegetables such as carrots, spinach, broccoli, mangoes, turnips, and sweet potatoes.	[32,65,79,82,83]
Vitamin E	Upper GI cancers, Colon cancer, HCC	15 mg	Vegetable oils (cotton seed oil, wheat germ oil, corn germ oil, and peanut oil). All green plants contain some concentration of tocopherol but some green leafy vegetables and rose hips contain more than wheat germ.	[32,79,82,83,84]
Vitamin C	Intestinal metaplasia, HCC	90 mg	Fruits (especially citrus fruits) and vegetables (especially peppers andpotatoes).	[32,85,86,87]
Zinc	Esophageal tumors, Gastric cancers, Colon cancer, HCC	11 mg	Oysters, red meat, nuts, whole grains, poultry, and dairy products.	[32,88,89,90,91]
Selenium	Colon cancer, HCC	0.055 mg	Brazil nuts, seafoods, meats, grains, dairy products, eggs, and organ meats.	[32,92,93]
